# The sucker-like end-to-side arterial anastomosis for free flap in extremities reconstruction: a retrospective study of 78 cases

**DOI:** 10.1186/s13018-024-04597-z

**Published:** 2024-02-04

**Authors:** Liangkun Cheng, Yuzhong Tan, Liuchao Cui, Yun Zheng, Chenghe Qin, Lin Tian

**Affiliations:** 1 Department of Hand Microsurgery, Chongqing Great Wall Orthopaedic Hospital, Chongqing, 400016 China; 2 Department of Orthopedics - Traumatology, Nanfang Hospital, Southern Medical University, Guangzhou, 510515 China

**Keywords:** Microsurgery, End-to-side anastomosis, Free flap, Tissue reconstruction, Size discrepancy

## Abstract

**Background:**

The application of end-to-side (ETS) anastomosis for flap transfer poses challenges, particularly in cases of significant size discrepancy between the donor and flap arteries. Herein, a novel ETS anastomosis technique, termed “sucker-like ETS anastomosis”, is developed to mitigate and rectify such vessel discrepancies. This study aims to evaluate the efficacy of this technique in tissue defect reconstruction through free flap transfer.

**Methods:**

Between September 2018 and March 2023, the medical records and follow-up data of 78 patients who underwent free flap transfer using sucker-like ETS anastomosis for significant artery size discrepancies were collected and retrospectively analyzed.

**Results:**

Among the 78 cases that received free flap transfer, the range of artery size discrepancy (flap artery vs donor artery) was 1:1.6–1:4 (mean: 1:2.5). Following anastomosis with the sucker-like ETS technique, 75 cases achieved flap survival without requiring additional surgical intervention, yielding a one-stage success rate of 96.2%. Three cases experienced post-operative venous crises, with two cases surviving after vein exploration and one case undergoing flap necrosis, necessitating a secondary skin graft. Seven cases faced delayed wound healing but eventually achieved complete healing following dressing changes. No arterial crisis was observed during hospitalization. With an average follow-up of 13 months, the surviving flaps exhibited excellent vitality without flap necrosis or pigment deposition. Overall, the application of sucker-like ETS arterial anastomosis for flap transfer resulted in a high overall surgical success rate of 98.7% (77/78).

**Conclusion:**

The application of sucker-like ETS anastomosis for free flap transfer is highly effective, particularly in cases with significant size discrepancy between the recipient and donor arteries.

## Background

The prevalence of soft tissue defects has surged with the increasing incidents of traffic trauma and surgical procedures. Post-debridement or tumor resection, the reconstruction of tissue defects is commonly accomplished through direct sutures, skin grafts, or various flap transfer techniques, selected based on the wound size and the exposure of bones, tendons, or vessels [1]. Due to its versatile functionality, free flap transfer has emerged as the gold standard for severe wound defect reconstruction [2]. This method has now reached a high level of maturity, boasting a reported survival rate exceeding 95% [3–5]. Despite the low failure rate, complications in flap transfer significantly impact limb function and appearance, leading to extended hospital stays and increased economic expenses.

Vascular anastomosis is recognized as one of the most important factors for flap survival during microvascular surgery [1]. The failure of a free flap transfer, as reported, is largely attributed to the unfavorable arteriovenous thrombosis formation caused by an unsuccessful vessel anastomosis [3, 6]. Therefore, selecting an appropriate anastomosis method is crucial for the success of transferred free flaps. Conventionally, end-to-end (ETE) anastomosis is preferred for arterial anastomosis due to its ease of operation, strong reliability, and versatility [7]. However, the technique is unsuitable in limbs with a significantly repaired blood supply, since the dissociation of the donor artery sacrifices the downstream inline perfusion [8]. This vulnerability usually renders the distal extremity susceptible to necrosis or frostbite, particularly in winter. In such cases, end-to-side (ETS) anastomosis is an alternative technique, since it avoids the ligation of the donor vessels by securing the flap artery to the wall of the donor artery. In extremities reconstruction, the ETS technique has been reported to yield a higher success rate than the ETE counterpart [9]. Nevertheless, the complexity of ETS anastomosis sharply increases with significant size discrepancies between the donor and flap arteries, especially when the size ratio exceeds 2:1 (or 1.5–3:1, according to a previous study [5]). Operations in such situations are undoubtedly complex and time-consuming. Furthermore, an unsatisfactory anastomosis in arteries with size discrepancies often leads to flow separation and vortex formation [5, 10, 11], potentially resulting in thrombus formation and flap necrosis. These challenges inevitably limit the application of ETS anastomosis in arteries with significant size discrepancies. To date, although various modified anastomosis techniques have been adopted to overcome these challenges [3, 7, 12], such as altering the anastomosis angle of the donor and recipient vessels or enlarging the size of the stoma on the recipient vessel, the scope and therapeutic efficacy of these modified techniques remain limited in the literature.

Herein, a novel ETS anastomosis technique, termed "sucker-like ETS anastomosis", is developed to correct vessel discrepancies. Briefly, this technique involves pruning the stoma of the recipient artery into a sucker shape and anastomosing the donor and recipient arteries at a more oblique angle. In clinical practice, we observed that the sucker-like ETS anastomosis technique achieved a high success rate and involves straightforward operational procedures. The objectives of this retrospective study are to demonstrate our experience in this modified ETS anastomosis technique and assess its efficiency in severe tissue defect reconstruction.

## Materials and methods

The medical records and follow-up data of 78 patients who underwent free flap transfer using modified sucker-like ETS anastomosis for significant artery size discrepancies were collected and retrospectively analyzed, with data collected from September 2018 to March 2023. The patients' demographic characteristics are summarized in Table 1. Briefly, there were 53 male and 25 female patients, with ages ranging from 12 to 75 years (mean: 51.5). Among these patients, 27 underwent upper limb tissue defect reconstruction, while 51 underwent lower limb soft tissue defect reconstruction. In terms of the etiology, tissue defects in 50 cases were caused by trauma, while the other cases were caused by burns (*n* = 11) and infections (*n* = 17). In all cases, the indication for sucker-like ETS arterial anastomosis was decided by a senior surgeon and the surgery was carried out by a minimum of three experienced surgeons (including one senior surgeon). The indication for surgery included the significant exposure of bones, blood vessels, nerves, and ligaments that could not be sutured directly or transferred with an island flap. The medical records of the 78 patients were reviewed to determine the defect location, surgical duration, type of flap used, anastomotic vessels, and post-operative complications.

**Table 1:** The demographic characteristics of 78 patients

Characteristics	Total/Mean
No. of patients	78
*Age (years)*	
Mean	51.5
Range	12–75
*Location*	
Upper extremity	27
Lower extremity	53
*Cause*	
Trauma	50
Burns	11
Infections	17

Before surgery, all patients were informed regarding the details of the surgery, with written informed consent obtained from the patients or legal guardians pre-operatively. The protocols of this retrospective study were approved by the Ethical Committee of Chongqing Great Wall Orthopaedic Hospital.

### Surgical procedure

**Fig. 1:**
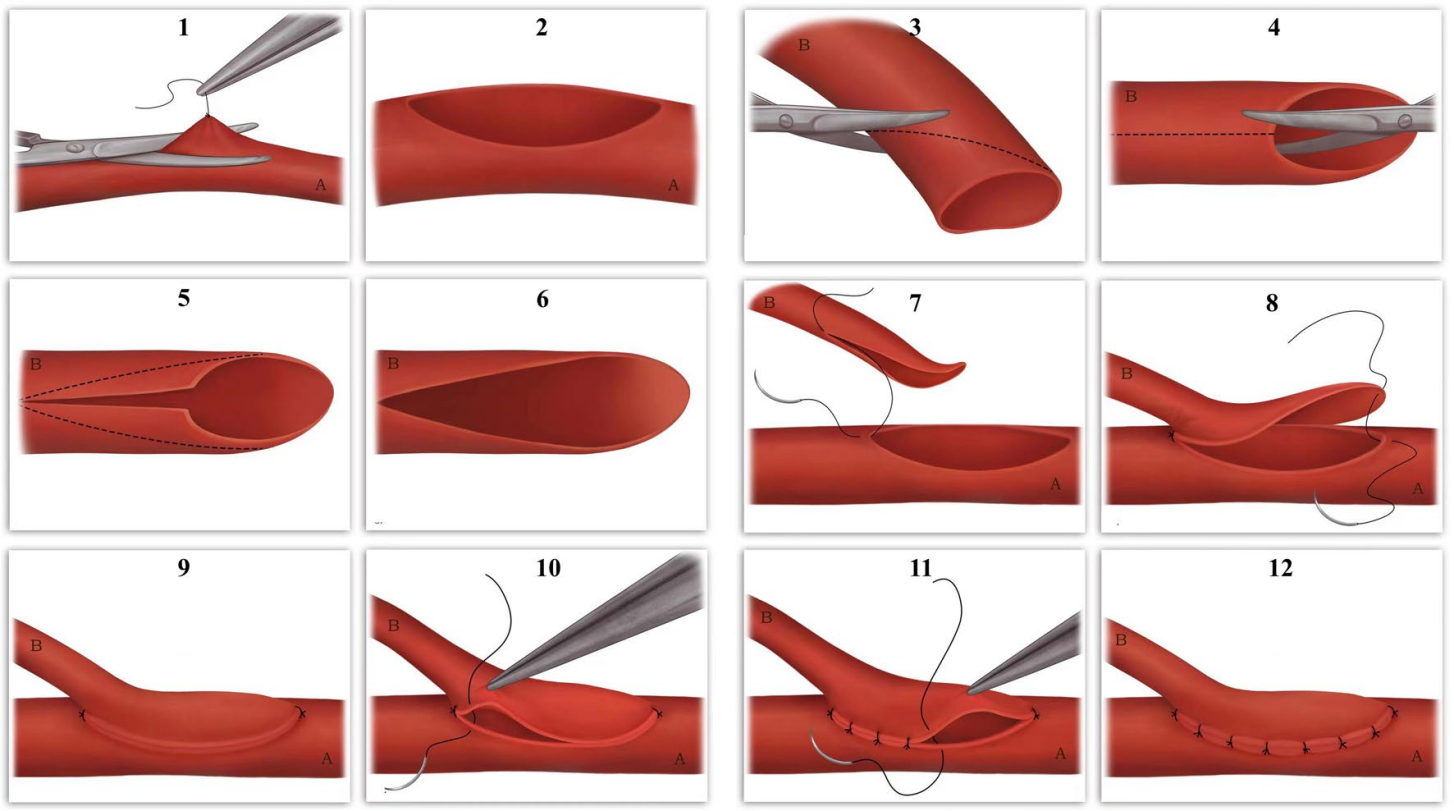
Diagram of the modified sucker-like ETS technique. (1–2): The vesiculotomy carried out on the wall of the donor artery. (3–6): The stoma of the recipient artery is cut to form a sucker-like appearance. (7–12): Sucker-like ETS arterial anastomosis performed to connect the donor and recipient arteries. **A** The donor artery. **B** The flap artery

**Fig. 2:**
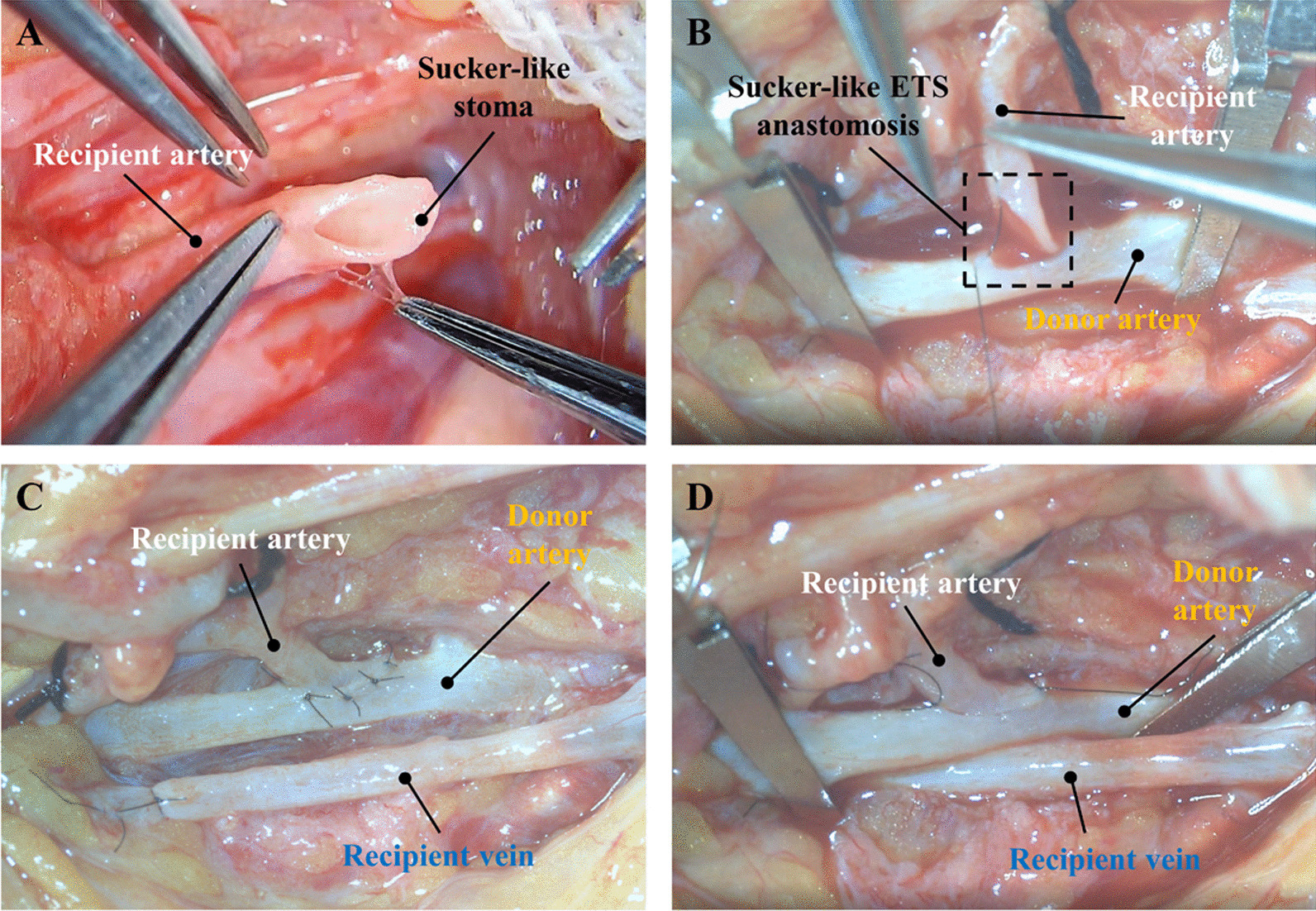
Representative intra-operative views of the modified sucker-like ETS technique

**Fig. 3:**
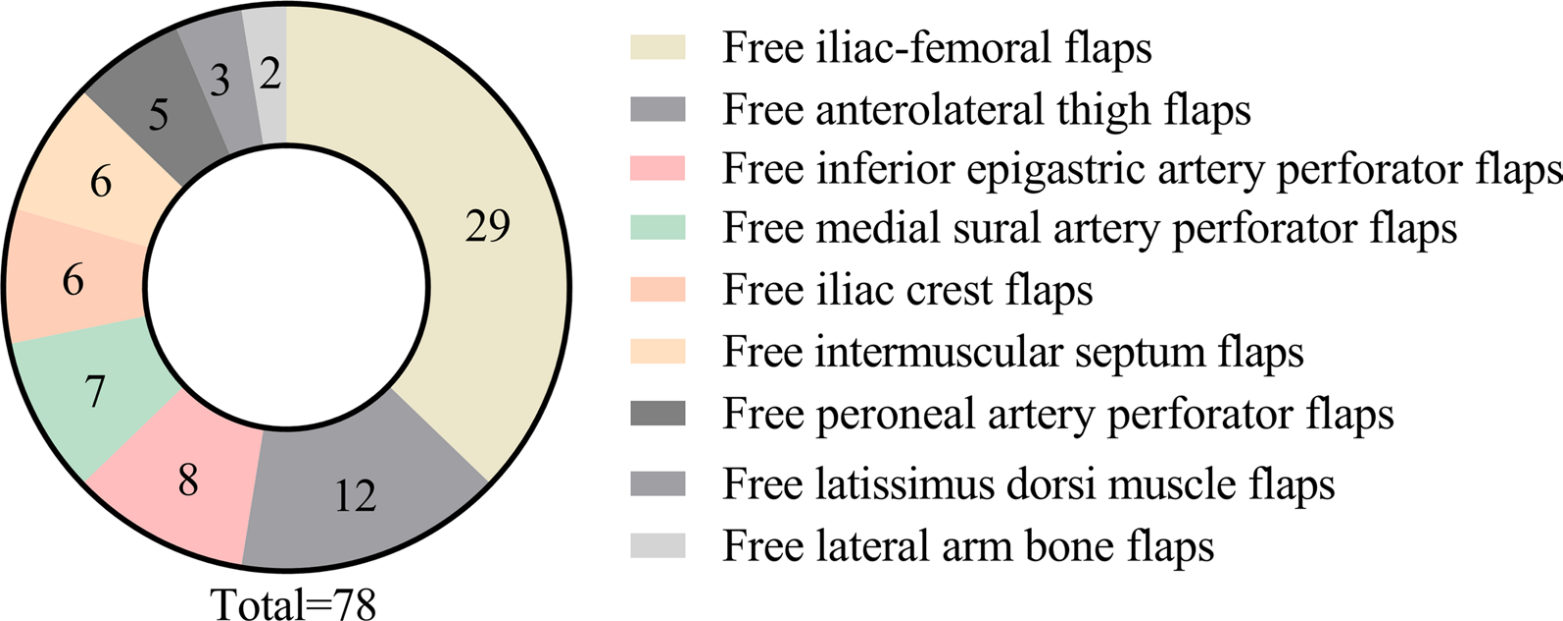
The types of free flap utilized in this study

**Fig. 4:**
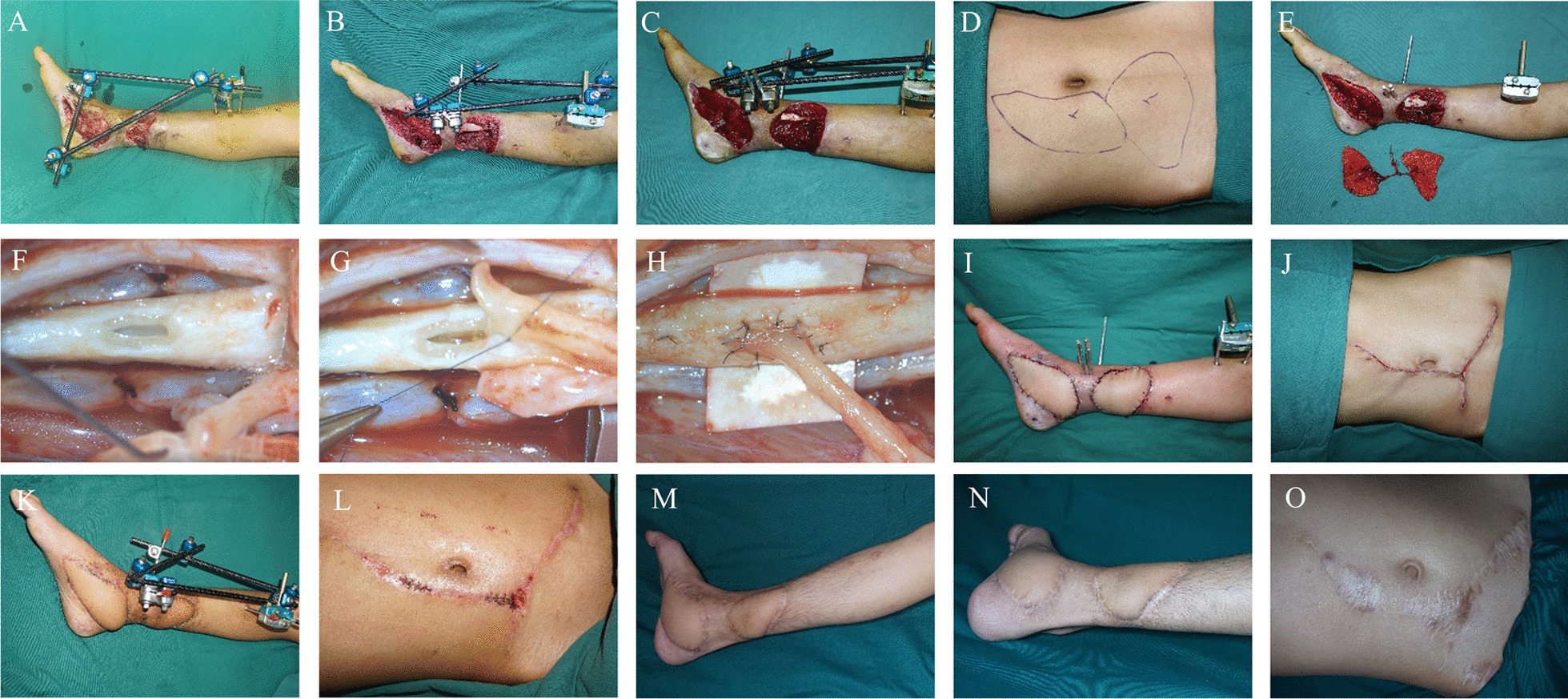
Indication (**A**), before (**B**), during (**C**–**I**) and after (**F** and **J**) surgery, and the result (**K**–**O**) of a patient subjected to a free flap transfer using the sucker-like ETE anastomosis technique

**Fig. 5:**
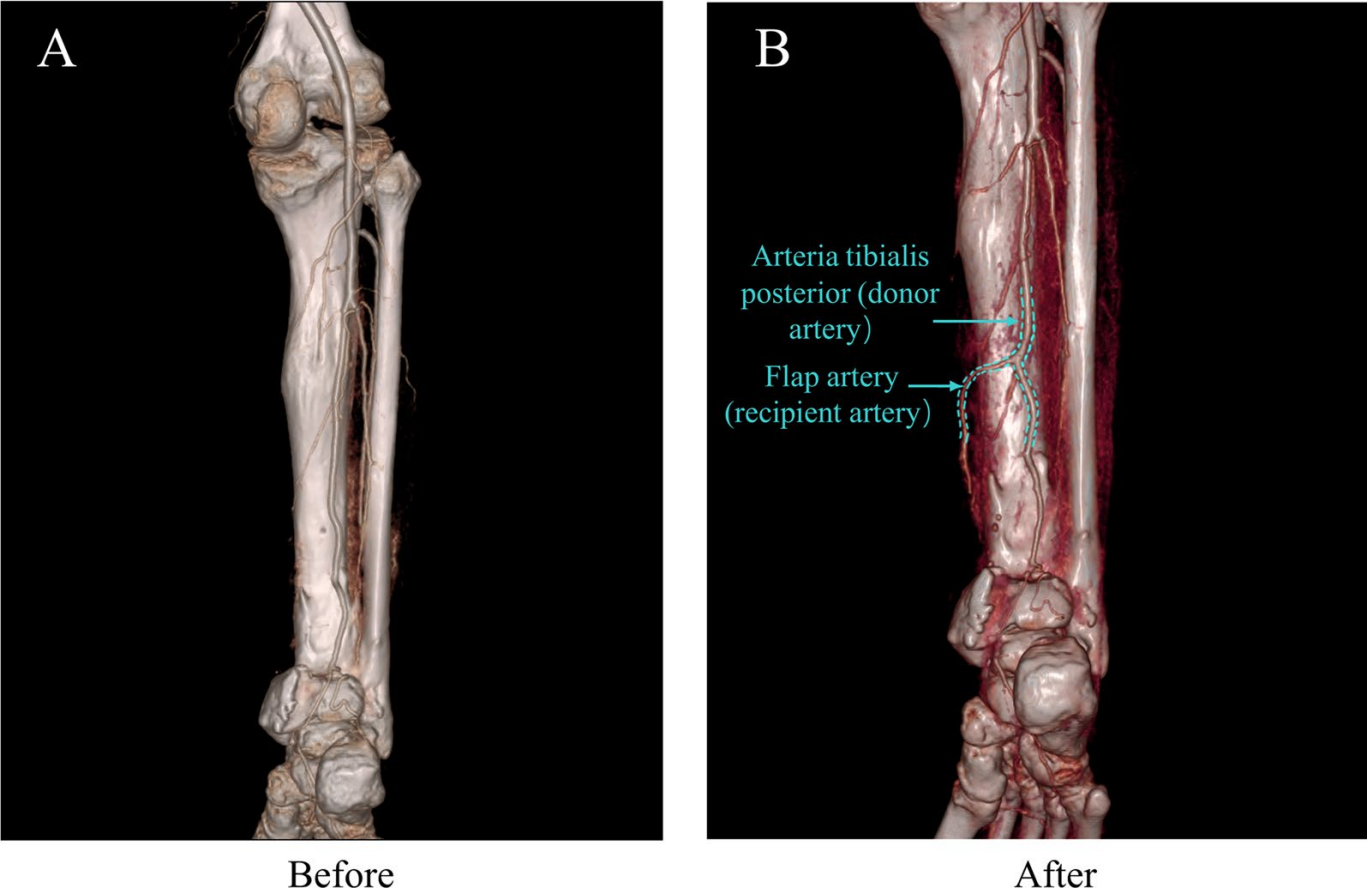
CTA examination of the donor and recipient arteries, where the images showed good patency in the anastomosis site post-surgery

The modified sucker-like ETS arterial anastomosis was performed following a standardized procedure. First, the wound was thoroughly debrided, and hemostasis was achieved. The debridement criteria included the presence of fresh, bleeding soft tissue or the observation of the “Paprika sign” in bones [13, 14]. After debridement, a lengthened incision was made at the proximal end of the wound (or an arc-shaped incision on healthy skin near the wound’s lateral side) to expose the donor vessels. Meanwhile, the free flap was harvested from the donor site, with one-stage closure of the wound areas in the majority of cases (or secondary closure in a few cases). The free flap was then placed over the wound and partially sutured to cover the wound and stabilize the flap. The vascular pedicle of the free flap was positioned in parallel to the donor vessels, followed by a meticulous dissection of the vessels in both the donor and recipient pedicles under a microscope. Prior to anastomosis, the flap (recipient) artery was cut at an oblique angle of 45–60° and longitudinally opened on one side, forming a suction cup-like shape. This design aimed to increase the diameter of the recipient stoma, surpassing that achieved by conventional ETS anastomosis (Figs. 1-1-1-6, 2A). Subsequently, a lateral stoma, equal in size to the flap artery stoma, was created in a suitable position on the wall of the donor artery (Fig. 1-2). After heparin saline irrigation, the flap artery was sutured to the donor artery along the direction of blood flow at an acute angle in the 25–40° range. This anastomosis angle is comparatively shallower than the conventional one utilized in the ETS anastomosis, with the aim of enhancing the blood flow (Figs. 1-7-1-12,  2B, C). The anastomosis began with the connection of the flap artery to the donor artery by suturing the distal-most and proximal-most points using the two-point fixation technique at an angle of 180° (Figs. 1-7-1-9, 2B). Subsequently, the anterior and posterior walls were sutured using the interrupted suture technique (Figs. 1-9-1-12, 2C, D).

Following arterial anastomosis, one-to-four of the accompanying veins with appropriate length in the flap area were dissociated and anastomosed with the recipient veins using the ETE or ETS anastomosis method.

### Post-operative management

After surgery, bed rest for 5–7 days was advised, accompanied by the elevation and immobilization of the affected limb. The post-operative regimen included infection control, anticoagulation, and spasm management. Smoking and alcohol consumption were strictly prohibited, and heating lamps were utilized for warmth. Continuous monitoring of the flap circulatory changes was carried out, with the prompt intervention of any anomalies. Routine post-operative pain relief medications were administered to establish a pain-free environment, aiming to promote patient comfort and relaxation.

The main outcomes of focus were the post-operative complications (e.g., vascular crisis) during the hospitalization period and the flap survival rate, flap failure rate, and secondary management during the follow-up phase. In the present study, flap failure is defined as a non-viable free flap necessitating subsequent flap transfer or skin-grafting for definitive soft tissue coverage.

## Results

The patients' characteristics and surgical details are presented in Table 2, while the flap types utilized for tissue reconstruction are presented in Fig. 3. As depicted in Table 2, the mean surgical period for a free flap transfer was 5.5 h (range: 4.5–7.5) and the mean ischemia time for the free flap was 1.8 h (range: 1.0–3.2). The average free flap area employed in our study was 160 cm^2^ (range: 8–260). Regarding to the size of the anastomosed arteries, the mean diameters of the flap arteries and the donor arteries were 1.0 mm (range: 0.5–2.5) and 2.0 mm (range: 0.8–4.5), respectively, yielding a mean size discrepancy of 1:2.5 (range: 1:1.6–1:4) between the two artery types.

**Table 2:** The outcomes of 78 patients during hospitalization and follow-up

Outcomes	Number
Surgical time	5.5 h (4.5–7.5 h, range)
Ischemia time	1.8 h (1.1–3.5 h, range)
Flap size (cm^2^)	160 cm^2^ (8–260 cm^2^, range)
Flap (recipient) artery (diameter, mm)	1.0 mm (0.5–2.5 mm, range)
Donor artery (diameter, mm)	2.0 mm (0.8–4.5 mm, range)
Size discrepancy (fold)	1:2.5 (1:1.6–1:4, range)
Num. of anastomosed veins	3 (1–4, range)
Vessel crisis	3 (3.8%, 3/78)
Flap failure	1 (1.2%, 1/78)
Delayed wound healing	7 (9.0%, 7/78)
One-stage flap survival	75 (96.2%, 75/78)
Overall flap survival	77 (98.7%, 77/78)
Reshaping surgery	12 (15.4%, 12/78)

In total, 78 free flaps in 78 patients were included and retrospectively analyzed, with 75 cases achieving survival without any additional surgical intervention, resulting in a one-stage success rate of 96.2% (75/78). Despite receiving double vein anastomosis, three cases (3.8%, 3/78) experienced venous crisis during the hospitalization period, in which two cases (2.6%, 2/78) were salvaged after venous exploration and one case (1.3%, 1/78) still presented with necrosis following post-surgery intervention. Nevertheless, this one case healed successfully through times of vacuum sealing drainage (VSD) coverage and a splint-thickness skin-grafting. Seven cases suffered from delayed wound healing, but eventually achieved complete healing after dressing change. In terms of the modified ETS technique, the anastomoses demonstrated superior arterial patency, and thus, no arterial crisis was observed in any of the flaps.

Throughout a mean follow-up period of 13 months (range: 3–42), the flaps in 77 patients, including those who underwent venous crisis exploration, exhibited favorable outcomes with no pigmentation changes. Notably, no instances of flap necrosis or infection were detected in these cases. Additionally, 12 patients (15.4%) underwent secondary debulking and reshaping surgery due to the swollen appearance of the flaps. Overall, with the application of the modified ETS anastomosis technique, 98.7% (77/78) of the cases achieved a satisfactory outcome, defined as successful flap survival. This high success rate underscores the effectiveness and positive clinical impact of the modified sucker-like ETS anastomosis in tissue defect reconstruction.

### Case report

A 16-year-old male was hospitalized for 10 days following the mechanical compression of his right lower limb. The wound dimensions on the distal medial aspect of the right calf measured approximately 15 × 8 cm, and on the medial aspect of the right foot around 15 × 5 cm, as depicted in Fig. 4A. Upon admission, debridement and VSD coverage were conducted on multiple occasions until the wound exhibited fresh granulation tissue (Fig. 4B, C). Pre-operative computed tomography angiography (CTA) was employed to identify the perforating branch of the inferior abdominal artery (Fig. 5A) and design the flaps (15 × 8 cm, 15 × 6 cm) (Fig. 4D). The perforator branch was subjected to a sucker-like ETS anastomosis with the posterior tibial artery (Fig. 4E, F), and the microscopic ETS anastomosis procedures (Fig. 4G–I) culminated in one-stage wound healing (Fig. 4J–L). Follow-up at 21-months post-surgery revealed that the skin flaps exhibited favorable sensation and texture, with no indications of flap deterioration, ulceration, or donor area keloid formation (Fig. 4M–O). Post-operative CTA examination showed the anastomosed arteries had good patency (Fig. 5B).

## Discussion

In the process of free flap transfer, the recipient and donor vessels can generally be anastomosed in either an ETE or ETS manner. The main distinction between the two techniques lies in the fact that an ETE anastomosis requires the sacrifice of a major artery in order to maintain the flap's blood supply, while placing heightened demands on the diameter matching between the vessels in the donor and recipient sites [7, 15, 16]. These disadvantages limit the application of the ETE technique in the reconstruction of extremities with compromised blood supply. In contrast, the ETS technique avoids the sacrifice of vital vessels and the requirement for strict size matching between two vessels, rendering it a more suitable method for the reconstruction of extremities with insufficient blood supply. Additionally, as the circular or helical smooth muscles on the wall of the donor vessels have been severed, the application of the ETS technique reduces the risk of anastomosis occlusion caused by the spastic contraction of the smooth muscles [1]. These advantages make the ETS technique more suitable for extremities reconstruction, although the procedure can become extremely challenging when a significant size discrepancy exists between the donor and recipient arteries. The risk of thrombus formation also sharply increased if conventional ETS anastomosis was performed in such situations. To overcome the shortcomings of conventional ETS anastomosis, a modified sucker-like ETS anastomosis technique was thus designed by enlarging the stoma of the recipient artery and connecting the arteries at a tilted angle. After being performed in practice, this new technique yielded a high flap success rate (98.7%) and a relatively low complication rate (1.3%) in patients undergoing free flap transfer. The reasons for this high success rate could be attributed to the following modifications: First, a sucker-type shape in the recipient artery effectively increases the diameter of the anastomosis site, while the adjustment of the anastomosis angle results in more efficient hemodynamics, both of which reduce the formation of vortices and lead to a higher flow patency and improved blood supply when compared to the conventional ETS technique. In addition, a wider slit on the wall of the donor artery also allows easy vesiculotomy and reduces the risk of anastomotic narrowing [17]. Collectively, these modifications enhance the efficacy of the suction-type ETS technique, serving as a valuable complement to the conventional ETS anastomosis approach.

Pedicle thrombosis formation remains a major challenge for the success of ETS anastomosis [1]. Conventionally, the formation of pedicle thrombosis is closely related to the size discrepancy between the anastomosed vessels, which usually results in a notably decreased patency rate and impaired hemodynamics [18]. Previous studies have reported that thrombosis formation is the most common cause of flap failure, with a thrombosis rate of 1.15–11.00% in arteries and 0.00–11.20% in veins [1, 19, 20]. In the present study, no arterial thrombosis formation was recorded following a modified sucker-like ETS anastomosis. In veins, thrombosis formation was detected in four cases, yielding a re-operation rate of 3.8% and a flap failure rate of 1.3%. While the thrombosis rate in veins was similar to other studies, the incidence of thrombosis in arteries was relatively lower. These outcomes, as mentioned above, were largely attributed to an enlarged anastomosis size as well as a more tilted angle of anastomosis (see Fig. 1) when using the sucker-like ETS anastomosis. Such modifications effectively avoid the size mismatch between two arteries in ETE anastomosis, while leading to improved patency and more stable hemodynamics than the conventional ETS anastomosis. This conclusion is also supported by a previous study where *Monsivais* grafted the veins of different diameters to the partially resected femoral artery, aiming to evaluate the influence of size discrepancy on the vessel patency. According to his research, when the grafted vein to artery ratio was 1:1, the patency rate could be retained at 90%; and when the ratio was 0.75:1, the patency rate could be retained at 80%. However, when the ratio of vessels was decreased to 0.25:1, the patency rate sharply decreased to 20% [18].

Besides the thrombosis formation, the surgery time of sucker-like ETS anastomosis was another focus of concern, since prolonged ischemic time and surgery time are another potential factor leading to flap necrosis. Interestingly, although more procedures are required to create a sucker-like stoma, the surgery time and the ischemia time in our study were not significantly prolonged compared to the time required for ETS anastomosis reported in former studies [17]. These results might be explained by the easier vascular anastomosis following the enlargement of the anastomosis stoma and a wider slit created on the wall of the donor artery. Collectively, the above-mentioned results indicate that the sucker-like anastomosis is effective for vessels with size discrepancy, while not prolonging the duration of surgery.

The parachute ETS anastomosis technique is widely employed by cardiovascular and vascular surgeons for various types of bypass surgery. In prior studies by *Naoya Watanabe *et al., the microscopic parachute ETS (MPETS) anastomosis technique was utilized to address the diameter discrepancies in vessels [17, 21]. According to their account, MPETS shares similarities with our sucker-like anastomosis technique, involving the trimming of stomas in both donor and recipient vessels in order to mitigate the drawbacks arising from size mismatch. This methodology has demonstrated promising therapeutic outcomes, with the majority of flaps exhibiting viability when employing the MPETS technique for both arterial and venous anastomosis [17, 21]. In comparison with the MPETS technique, the sucker-like anastomosis technique investigated in the present study has a number of distinct advantages: (1) While MPETS widens the stoma of the flap artery, the obtuse angle on both sides remains inadequately trimmed, potentially leading to an uneven incision. Conversely, in sucker-like ETS anastomosis, the flap artery stoma necessitates precise trimming into a sucker to achieve optimal patency and a seamless connection. (2) Compared to the “slit window" in MPETS, the sucker-like ETS anastomosis prunes the donor artery stoma to an oval shape, which tends to result in in a smoother connection and is less prone to thrombus formation. (3) The MPETS technique imposes stringent requirements for a specialized double-needle microsuture, necessitating double-needle stitches, while in sucker-like ETS anastomosis, a single needle suture suffices. 4) The operational procedures of MPETS anastomosis are particularly intricate, involving the initial fixation of five bundles of parallel lines at the beginning of the suture; this demands precise coordination from the surgical assistant, with careful attention necessary to avoid suture knotting. In contrast, sucker-like ETS anastomosis requires a simpler single needle intermittent suture, thus lowering the technical demands on both the surgeon and the assistant. 5) The intricate stitching method of MPETS anastomosis results in more time-consuming procedures. Conversely, the procedures for the sucker-like ETS technique are comparatively streamlined, leading to a simpler and faster anastomosis process.

While the sucker-like ETS anastomosis exhibits definite advantages, as per other anastomosis methods, it requires surgeons with proficient microsurgical skills and extensive clinical practice. In our experience, to perform a sucker-like ETS anastomosis, attention to the following aspects is required: (1) As the patency of the vessels is critical for flap survival, it is essential to confirm the patency of the donor vessels both pre- and intra-operatively. (2) Evaluation of the length of the vascular pedicle of the harvested flap is necessary to avoid vascular shortages during anastomosis. (3) When creating the side stoma in the donor vessels, the full vessel wall layers should be completely removed, especially in cases with vascular intimal layer separation. The stoma should not exceed half of the vessel's circumference (1/3 of the vessel's circumference is preferred), and the stoma should preferably be created as an elongated elliptical shape. (4) When creating an oblique stoma, a longitudinal incision on the blunt-angle side of the stoma should be performed to acquire an ideal sucker-like-shaped stoma, with the aim of enlarging the area of the anastomosis (larger than the oblique stoma in standard ETS anastomosis). (5) Proper alignment of the vascular pedicle should be maintained with moderate tension, and tension, kinking, or twisting avoided to ensure that the vascular pedicle is not compressed. (6) Post-operatively, standard procedures for infection control, anticoagulation, and anti-spasm treatment need to be carried out.

There are a number of limitations to this study. First, the size of the study sample is relatively small, and therefore, future work should include a larger sample. Moreover, the outcomes are not compared with those of other anastomosis techniques, and thus, a comparative study is necessary to confirm our results. Finally, this investigation's retrospective nature means that only limited information was available, thus reducing the credibility of our study.

## Conclusion

The present study indicates that the sucker-like ETS arterial anastomosis is an effective technique in the reconstruction of severe tissue defects in extremities, which could be a valuable complement to the conventional ETS anastomosis technique. We recommend the preferential use of sucker-like ETS anastomosis for arteries with significant size discrepancies, as it can provide favorable results in free flap transfer. However, a randomized control study with a larger number of cases would provide more convincing results.

## Data Availability

Data and materials will be available on reasonable request.

## Abbreviations

ETE: End-to-end
ETS: End-to-side
CTA: Computed tomography angiography
VSD: Vacuum sealing drainage
MPETS: Microscopic parachute ETS anastomosis
